# The Role of Methylation in Neurodegenerative Diseases: Insights From a Bibliometric Study

**DOI:** 10.1002/brb3.70732

**Published:** 2025-08-04

**Authors:** Jianwei Xu, Junhou Lu, Tao Kuang, Dongyan Wang, Zhihui Dong, Maoqiong Chen, Can Liu, Guo Ge, Tianhong Yuan, Zhen Qin

**Affiliations:** ^1^ Center for Tissue Engineering and Stem Cell Research Guizhou Medical University Guiyang China; ^2^ School of Basic Medical Guizhou Medical University Guiyang China; ^3^ Department of Neurosurgery Second Affiliated Hospital of Zunyi Medical University Zunyi China; ^4^ Clinical Laboratory First People's Hospital of Guiyang City Guiyang China; ^5^ Pediatrics Affiliated Hospital of Guizhou Medical University Guiyang China; ^6^ Guizhou Guizhong Biotechnology Co. Ltd Guiyang China; ^7^ Key Laboratory of Molecular Biology Guizhou Medical University Guiyang China; ^8^ Department of Pharmacology, School of Basic Medicine Guizhou University of Traditional Chinese Medicine Guiyang China

**Keywords:** bibliometrix, CiteSpace, methylation, neurodegenerative disease, VOSviewer

## Abstract

**Background:**

Neurodegenerative diseases are characterized by a progressive decline in neuronal function, posing a major challenge to understanding their molecular mechanisms. The role of DNA methylation in these diseases remains an area of research focus.

**Methods:**

A bibliometric analysis was performed using the Web of Science Core Collection (WoSCC) database, covering 3284 publications from January 1, 2000 to November 18, 2024. The search focused on articles related to methylation in neurodegenerative diseases, with queries limited to the “title” field. Only original articles and reviews in English were included. Data were analyzed using VOSviewer, CiteSpace, and Bibliometrix R packages to visualize trends, identify collaborative networks, and identify key topics, such as DNA methylation and Alzheimer's disease.

**Results:**

The analysis showed a steady increase in research output, with major contributions coming from the United States, China, and the United Kingdom. The University of California system, Harvard University, and University College London were leading institutions. Key journals such as the International Journal of Molecular Sciences, Nature, and Proceedings of the National Academy of Sciences were identified as having impact. Keyword analysis highlighted DNA methylation and Alzheimer's disease as prominent research topics.

**Conclusion:**

Despite limitations such as the lack of experimental validation and clinical evidence, this study highlights the growing interest in methylation research on neurodegenerative diseases and underscores the need for innovative efforts to identify novel therapeutic targets and biomarkers.

## Graphical abstract

This bibliometric analysis of 3284 articles from 2000 to 2024 explores global research trends, productive countries, institutions, and keyword hotspots in methylation and neurodegenerative diseases, with Alzheimer's disease and DNA methylation emerging as key research focuses.

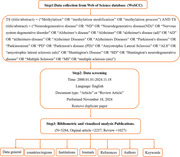

## 1 Introduction

Neurodegenerative diseases represent a significant challenge to public health, characterized by the progressive loss of neural cells leading to cognitive and behavioral impairments (Agnello and Ciaccio 2022; Dugger and Dickson 2017). These disorders, including Alzheimer's disease, Parkinson's disease, and amyotrophic lateral sclerosis, impose considerable burdens on patients and healthcare systems worldwide due to their complex etiology and the associated healthcare costs (2024 Alzheimer's Disease Facts and Figures 2024; Aamodt et al. 2024). While genetic mutations have been identified in some cases, the majority of instances are sporadic and linked to environmental risk factors, necessitating a deeper understanding of their underlying mechanisms (Goldman 2020; Clarimon et al. 2020; Verheijen and Vermulst 2024). Moreover, neuroinflammatory processes, particularly involving microglial cells, play a crucial role in the pathophysiology of these diseases (Gao et al. 2023).

Current research methods primarily focus on symptomatic management rather than addressing the molecular underpinnings of neurodegenerative diseases (Passeri et al. 2022). Accordingly, there is a pressing need for innovative research strategies aimed at identifying novel therapeutic targets and biomarkers that go beyond symptomatic management and address the underlying molecular mechanisms. Recent advancements in understanding epigenetic factors, particularly methylation, have opened new avenues for research in this domain (Yang et al. 2023; Younesian et al. 2022). Methylation alterations have been implicated in the regulation of gene expression linked to neurodegenerative pathologies, suggesting that epigenetic modifications may serve as integral components in the complex interplay of genetic and environmental factors contributing to disease progression (Berson et al. 2018; Basavarajappa and Subbanna 2021; Xu et al. 2021). A comprehensive exploration of methylation in the context of neurodegenerative diseases holds the potential to unravel the intricate molecular mechanisms involved and inform future therapeutic strategies.

The present study employs a bibliometric analysis to systematically evaluate the trends, influential contributors, and collaborative networks within the field of methylation research related to neurodegenerative diseases. This method allows for a quantitative assessment of the literature, providing insights into publication patterns and identifying key research outputs that can guide future studies. Utilizing comprehensive data retrieved from the Web of Science Core Collection (WoSCC), this analysis aims to delineate the landscape of research output over the past two decades, highlighting the evolution and growth trajectory of methylation studies in relation to neurodegenerative diseases.

The primary objective of this study is to assess the current state of methylation research in neurodegenerative diseases to guide future research and collaboration. By identifying leading institutions and countries contributing to this field, as well as the most influential authors and journals, this bibliometric analysis seeks to enhance the impact of methylation research on clinical applications. The ultimate goal is to deepen our understanding of the molecular mechanisms underlying neurodegenerative diseases.

## 2 Materials and Methods

### 2.1 Data Retrieval and Collection

In this study, we utilized the WoSCC as our data source, a comprehensive and authoritative database encompassing approximately 10,000 prestigious academic journals (Jiang et al. 2023). Our bibliometric analysis focused on methylation research in neurodegenerative diseases, based on publications retrieved from WoSCC. Our search spanned from January 1, 2000 to November 18, 2024. To enhance the precision of identifying relevant studies, we limited our search query to the “title” field. We included only original articles and review articles written in English during the screening process. The retrieval results were exported in plain text format and as tab‐delimited files, with the content set to “Fully Recorded and Cited References.”

### 2.2 Bibliometric and Visualized Analysis

Bibliometric analysis and visualization of the publications retrieved from the WoSCC were performed using VOSviewer (version 1.6.20), CiteSpace (version 6.3.R1), the Bibliometrix package, and an online bibliometric analysis platform (https://bibliometric.com/).

VOSviewer is a powerful visualization tool that enables us to import literature data from the Web of Science database and conduct an in‐depth analysis using its diverse data source compatibility, co‐occurrence analysis, clustering analysis, and timeline view functionalities (Arruda et al. 2022). By generating visualizations such as tag clouds, density plots, and network diagrams, VOSviewer helps us intuitively display research trends, keyword co‐occurrences, and collaborative relationships, thereby identifying research hotspots and key researchers within the field and their collaborative networks (J. Wang and Maniruzzaman 2022). In this research, VOSviewer was utilized to visually map the collaborative relationships among nations, institutions, and journals, as well as the co‐citation networks of keyword clusters.

CiteSpace is a widely recognized visualization analysis software in the field of bibliometrics (He et al. 2023). Its capabilities include co‐citation analysis, collaboration network analysis, and keyword co‐occurrence analysis, which enable us to identify core literature, research collaboration networks, and research hotspots (Z. Liu, Yu, et al. 2023). Additionally, its time series analysis feature allows us to track long‐term trends in research topics. The graphical visualization and interactive exploration functions enhance the depth of data mining (Synnestvedt et al. 2005). In our study, we utilized CiteSpace to detect institutions, references, and keywords that exhibited significant citation bursts. We generated clustering maps to visualize these entities and created timeline visualizations to chart the progression of references over the years.

Bibliometrix is an R package designed for bibliometric analysis of scientific literature, enabling data import from multiple databases (B. Liu, Zhou, et al. 2023). It offers a comprehensive set of analytical tools, including calculations of publication count, citation frequency, and the *h*‐index (a measure of research productivity and impact) (Xia et al. 2021). Additionally, it facilitates the construction of coauthorship and co‐citation networks, as well as multidimensional scaling, principal component analysis, and clustering analysis (Dai et al. 2023). We utilized Bibliometrix to create a collaboration map that illustrates partnerships between countries and regions, along with other analyses.

## 3 Results

### 3.1 Annual Publication Analysis

**FIGURE 1:**
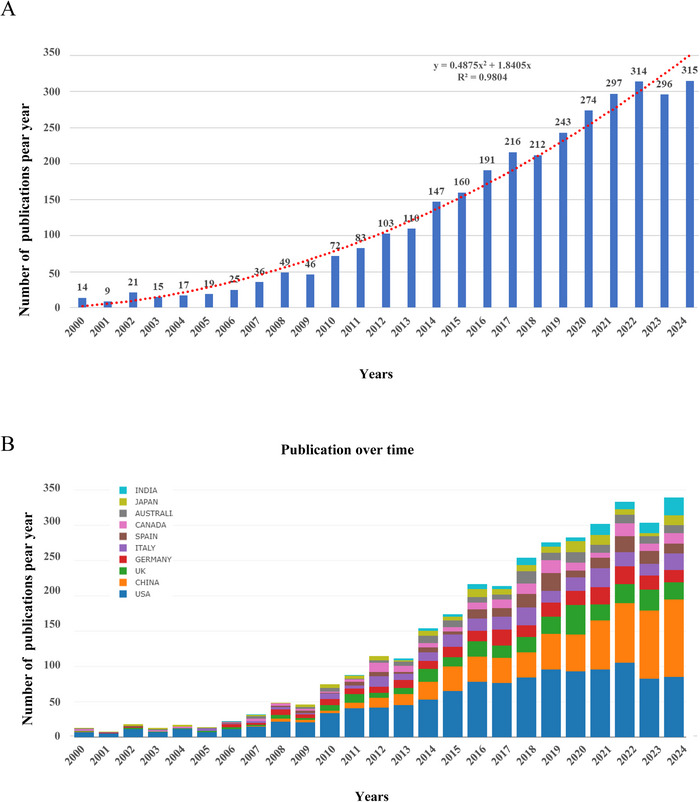
The annual number of publications on methylation research related to neurodegenerative diseases. (A) The number of publications by year and the curve fitting of the annual growth trend of publications in methylation research related to neurodegenerative diseases from 2000 to 2024 (*R* ^2^ = 0.9804). (B) Analysis of publication volume and growth trends in methylation research on neurodegenerative diseases from 2000 to 2024 for the top 10 countries/regions, represented in a bar chart indicating annual publication counts.

Analyzing the publication time and trend distribution of literature allows for a more intuitive judgment of the development speed and academic attention toward methylation research related to neurodegenerative diseases. In this study, a total of 3284 publications on methylation research related to neurodegenerative diseases were statistically analyzed (publications from 2024 were not fully included). The analysis included 2257 original articles (68.73%) and 1027 reviews (31.27%) after removing duplicates. The annual publication trends are depicted in Figure 1A. During the two decades under review, the total number of papers published each year has shown a consistent rise, exhibiting exponential growth. The equation *y* = 0.4875*x* ^2^ + 1.8405*x*, with a high coefficient of determination (*R* ^2^ = 0.9804), suggests a strong and consistent increase in the number of annual publications. These findings show that methylation research linked to neurodegenerative diseases is drawing more interest.

### 3.2 Distribution of Countries/Regions

According to the inclusion criteria, a total of 3284 articles were published by 3518 institutions from 95 countries and regions. Table 1 presents the top ten countries contributing to publications in the field of methylation research related to neurodegenerative diseases. It includes the total number of publications, total citations, average citations per publication, and total link strength (TLS) for each country. Among these countries, the United States leads with the highest number of articles, contributing 1195 articles (36.39%), followed by China with 679 articles (20.68%) and the United Kingdom with 299 articles (9.10%). The collective research output from these three nations accounts for over 66%, highlighting a significant concentration of interest in methylation studies related to neurodegenerative diseases. The publications from the United States also garnered the highest number of citations, totaling 64,155 citations, indicating their significant international influence. When comparing the academic contributions of China and Canada in the field of methylation research related to neurodegenerative diseases, some notable differences stand out. Although China ranks second in the total number of publications, its average citations per publication are relatively low. In contrast, Canada, despite ranking only seventh in the total number of publications, tops the list in average citations per publication. This suggests that Canada's research outcomes in this area have a higher level of impact and recognition, while China, despite producing a significant volume of research, has comparatively lower influence per paper. These differences may be due to research quality, level of international collaboration, novelty of research topics, and advancements in methodology. Figure 1B illustrates the total number of articles published by the top 10 countries from 2000 to 2024, showing that the number of publications in the field of methylation studies related to neurodegenerative diseases has grown rapidly.

**TABLE 1:** The top 10 countries/regions that contributed publications on methylation research related to neurodegenerative diseases.

Rank	Country/Region	Articles	Articles (%)	Citations	Average citations per publication	Total link strength
1	USA	1195	36.39	64155	54	825
2	China	679	20.68	15455	23	275
3	United Kingdom	299	9.10	17215	58	566
4	Germany	264	8.04	15472	59	420
5	Italy	263	8.01	10751	41	277
6	Spain	183	5.57	7859	43	272
7	Canada	173	5.27	11340	66	233
8	Australia	149	4.54	7467	50	176
9	Japan	138	4.20	4568	33	81
10	India	107	3.26	2881	27	65

**FIGURE 2:**
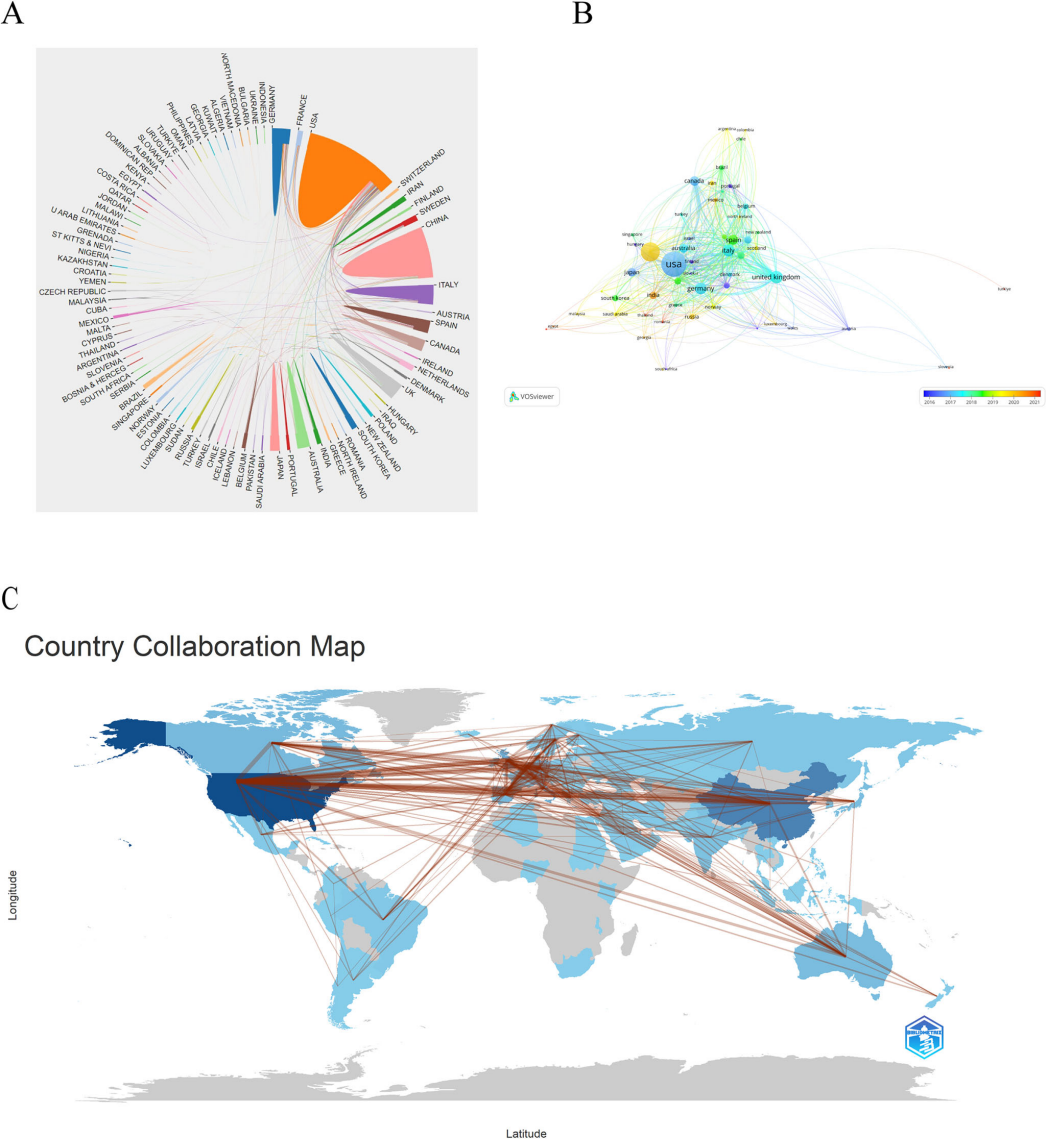
Contributions of countries/regions. (A) The bibliometrics online analysis platform (http://bibliometric.com/) creates a network of collaboration among countries/regions. The larger the area, the more articles the country/region publishes. Lines indicate connections between countries and regions. (B) The VOSviewer timeline visualization map depicts interactions among 57 countries and districts with more than five publications, where the node size represents the number of articles contributed by each country or district (C), Geographical distribution map of global collaborations among countries and regions in the field of methylation research related to neurodegenerative diseases.

We utilized the online bibliometric analysis platform (https://bibliometric.com) to analyze the significance of countries within collaborative networks, as depicted in Figure 2A. The United States emerged as the most influential, with a more active role than any other nation, followed closely by China, Germany, the United Kingdom, and Italy. Notably, the United States has established cooperative ties with the majority of countries globally. Based on the coauthorship analysis using VOSviewer software, countries were categorized into different clusters, with only those having a minimum of five publications included in the study. Out of 95 countries/regions, 57 qualified for visualization, as shown in Figure 2B. Among these, the top five countries with the highest TLS were the USA (TLS = 825), the United Kingdom (TLS = 566), Germany (TLS = 420), the Netherlands (TLS = 307), and Italy (TLS = 277), reflecting their significant academic influence and collaborative engagement in the field of methylation research related to neurodegenerative diseases. Furthermore, while the United States and European countries have had earlier publication dates, recent years have seen a surge in publications from Asian countries, including China, Thailand, and India. Figure 2C presents a geographical distribution map of global collaborations among countries and regions in the field of methylation research as it pertains to neurodegenerative diseases.

### 3.3 An Analysis of the Most Productive Institutions

**FIGURE 3:**
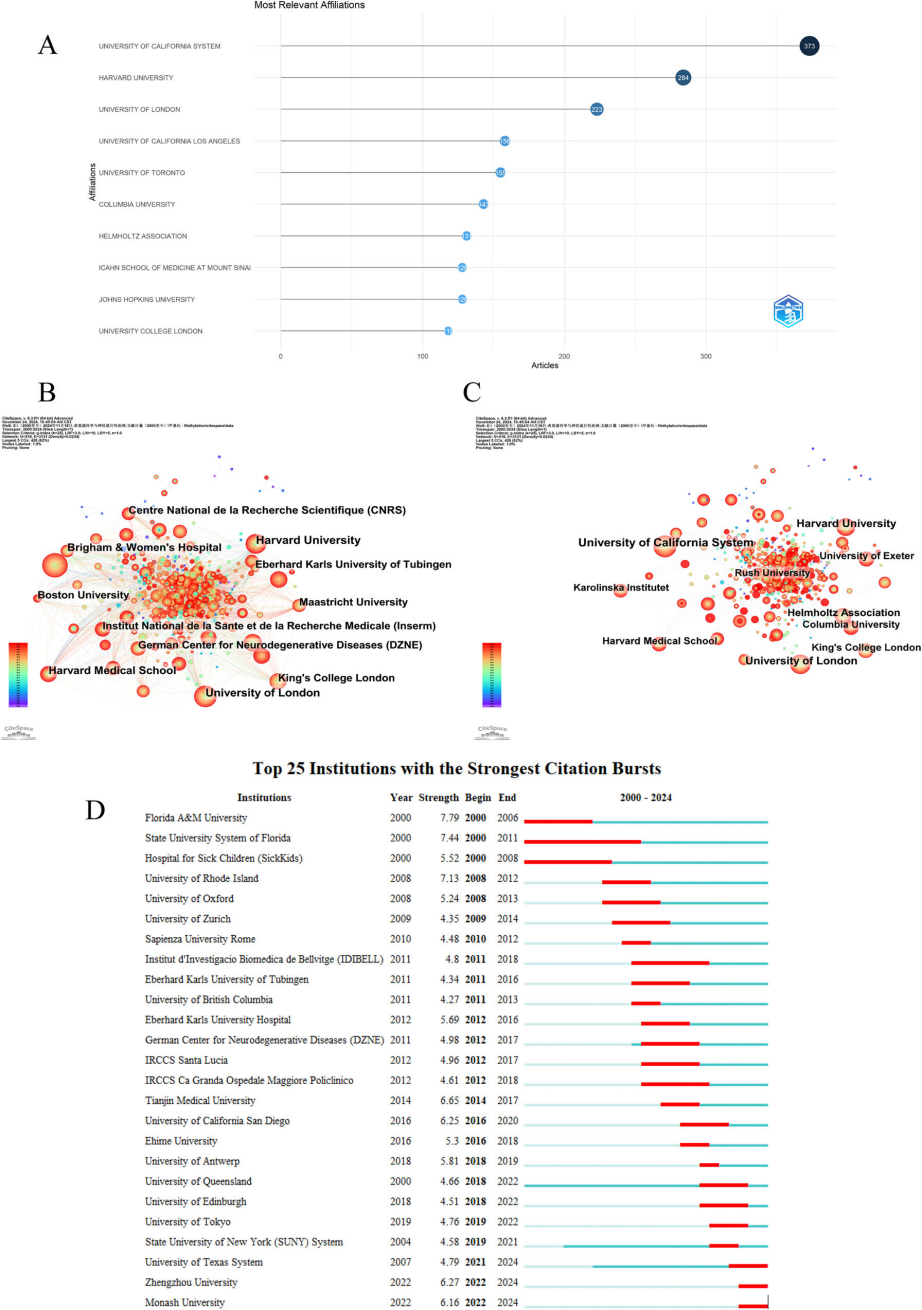
Contributions of countries/regions. (A) The top 10 affiliations with the most publication output in the field of methylation research related to neurodegenerative diseases. (B) Global collaborative network map in the field of methylation research related to neurodegenerative diseases (2000–2024), sorted by institutional collaboration degree using CiteSpace software. The size of the nodes represents the frequency of institutional collaborations, and the edges indicate relationships between institutions. (C) Global Collaborative Network Map in the field of Neurodegenerative Disease Methylation Research (2000–2024), sorted by institutional citation impact using CiteSpace software. Node size corresponds to the citation count, reflecting the scholarly influence of each institution, while edges represent collaborative ties between institutions. (D) The top 25 strongest bursts of institutions.

According to the data we retrieved, 3519 institutions participated in methylation research related to neurodegenerative diseases over the past 20 years. Figure 3A presents a ranking of the top 10 institutions with the most substantial publication output in the field of neurodegenerative disease methylation research, showcasing their considerable influence and academic prominence. The United States is home to six of these leading institutions, which underscores the country's dominant role in shaping the direction and progress of this scientific domain. Among the top 10, three institutions have made particularly noteworthy contributions by publishing over 200 papers each, reflecting their extensive research efforts and the breadth of their scholarly impact. The University of California System leads with an impressive 373 publications, indicating its significant role in advancing knowledge and driving innovation in this field. Harvard University follows with 284 publications, a testament to its longstanding reputation for academic excellence and its continuous commitment to producing groundbreaking research. The University of London, with 223 publications, also exhibits a strong presence in the field.

The global collaborative network map in the field of methylation research related to neurodegenerative diseases was created using CiteSpace software. It was sorted using “by degree” analysis to identify core institutions within the collaboration network. As shown in Figure 3B, the map illustrates that institutions, such as Harvard University, the University of London, Harvard Medical School, Brigham and Women's Hospital, and the French National Centre for Scientific Research (CNRS) have high node degrees. The size of the nodes represents the frequency of institutional collaborations, while the edges indicate the relationships between institutions. These institutions stand out due to their direct connections with multiple partners, indicating that they play a key role in collaborative research in this field. When we sorted by citation count, we found that the institutions within the University of California System, Harvard University, the University of London, the Helmholtz Association, and King's College London are the most prominent, as shown in Figure 3C. This indicates that their research outcomes have received widespread attention and recognition in the academic community. The high citation frequency of these institutions not only reflects the quality of their research in the field but may also indicate their key role in driving scientific progress. By further analyzing the collaboration patterns of these institutions, we can better understand how they enhance their research impact through cooperation. Figure 3D shows the top 25 institutions with the strongest citation bursts in the field of methylation research related to neurodegenerative diseases. The top three institutions were Florida A&M University (2000–2006, strength = 7.79), the State University System of Florida (2000–2011, strength = 7.44), and the University of Rhode Island (2008–2012, strength = 7.13). The University of Texas System, Zhengzhou University, and Monash University have also become increasingly engaged in this area of research in recent years.

### 3.4 Analysis of Journals and Cited Journals

**FIGURE 4:**
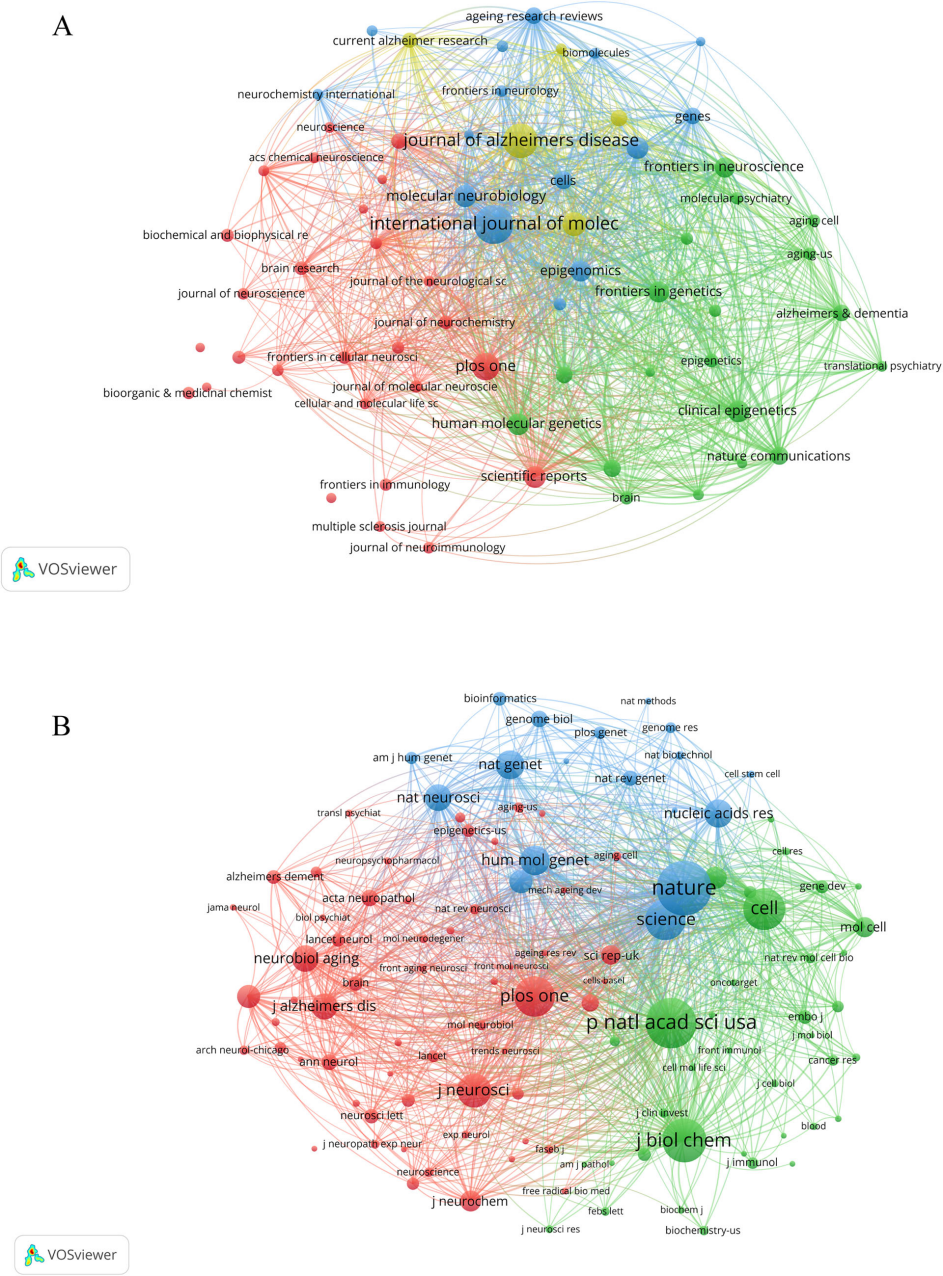
Analysis of journals in the field of methylation research related to neurodegenerative diseases. (A) Bibliographic coupling of journals with at least five papers in the field of methylation research related to neurodegenerative diseases. Each circle in the figure represents a journal, and the size of the circle indicates the number of publications output in that journal. (B) The network visualization map of journal co‐citation analysis was generated by VOSviewer, displaying only journals with at least 500 co‐citations. Each node represents a co‐cited journal, with different colors denoting distinct clusters, and the links between nodes indicate the co‐citation relationships among journals.

By conducting an in‐depth analysis of the characteristics, scope, and impact factors (IF) of journals in related research fields, we can uncover publishing trends, identify leading journals, and gain insights into the evolution of the academic publishing landscape. There are 949 journals active in the field of methylation research related to neurodegenerative diseases from 2000 to 2024. By setting a minimum of 10 documents per source, 66 of these journals meet the threshold, and a visualization of the sources was created (Figure 4A). In this analysis, each circle in the figure represents a journal, with the size of the circle indicating the number of publications in that journal and different colors denoting distinct clusters. This approach helps to reveal similarities in content and research topics between journals, reflecting the intrinsic connections and developmental patterns among scientific disciplines. The top 10 most active journals and the co‐cited journals are listed in Table 2. The top 10 most productive journals, which published a total of 548 articles representing 16.69% of the overall total, were predominantly categorized in Q1 and Q2. As shown in Table 2, the *International Journal of Molecular Sciences* led with 109 articles (3.32%, Q2), followed by the *Journal of Alzheimer's Disease* with 89 articles (2.71%), *PLOS One* with 61 articles (1.86%), and *Neurobiology of Aging* with 48 articles (1.46%). Among the Q1 journals ranked by publication volume, the three with the highest IF were *Clinical Epigenetics* (IF = 4.8, 40 articles, 1.22%), followed by *Molecular Neurobiology* (IF = 4.6, 42 articles, 1.28%), and *Scientific Reports* (IF = 3.8, 41 articles, 1.25%).

**TABLE 2:** The top 10 journals and the co‐cited journals that published documents on proteomics research related to neurodegenerative diseases.

Rank	Sources	Articles	Citations	Total link strength	IF (2023)	JCR (2023)	Co‐cited Journal	Co‐citations	Total link strength	IF(2023)	JCR (2023)
1	*International Journal of Molecular Sciences*	109	2130	69949	4.9	Q2	*Nature*	8035	687037	50.5	Q1
2	*Journal of Alzheimer's Disease*	89	3498	42207	3.4	Q2	*Proceedings of the National Academy of Sciences of the United States of America*	7274	613113	9.4	Q1
3	*Plos One*	61	3386	21463	2.9	Q1	*Journal of Biological Chemistry*	6125	498649	4	Q2
4	*Neurobiology of Aging*	48	2894	30375	3.7	Q2	*Cell*	5675	499583	45.6	Q1
5	*Molecular Neurobiology*	42	777	22970	4.6	Q1	*PLOS One*	5482	422835	2.9	Q1
6	*Human Molecular Genetics*	41	2097	23186	3.1	Q2	*Science*	5453	461845	44.8	Q1
7	*Scientific Reports*	41	845	17205	3.8	Q1	*Journal of Neuroscience*	4455	387175	4.4	Q1
8	*Clinical Epigenetics*	40	1243	28627	4.8	Q1	*Human Molecular Genetics*	3790	320525	3.1	Q2
9	*Frontiers in Aging Neuroscience*	40	1107	27367	4.1	Q2	*Nature Genetics*	3580	267258	31.8	Q1
10	*Epigenomics*	37	896	29479	3	Q2	*Nucleic Acids Research*	3459	235401	16.7	Q1

The co‐citation analysis of co‐cited journals, as depicted in Figure 4B and detailed in Table 2, reveals that “*Nature*” was the most frequently co‐cited journal, with 8035 citations and a TLS of 687,037, boasting an IF of 50.5. It is noteworthy that the majority of the journals included in this analysis are classified in the Q1 category. Figure 4B displays a network visualization map of co‐cited journals with over 500 citations each, organizing a total of 113 items into three distinct clusters. The representative journals in each cluster are as follows: *Journal of Neuroscience* and *PLOS One* are prominent in Cluster 1 (colored red), *Nature* and *Science* lead in Cluster 2 (colored blue), and *Proceedings of the National Academy of Sciences of the United States of America* and *Journal of Biological Chemistry* are significant in Cluster 3 (colored green).

### 3.5 Analysis of Productive Authors and Co‐Cited Authors

**FIGURE 5:**
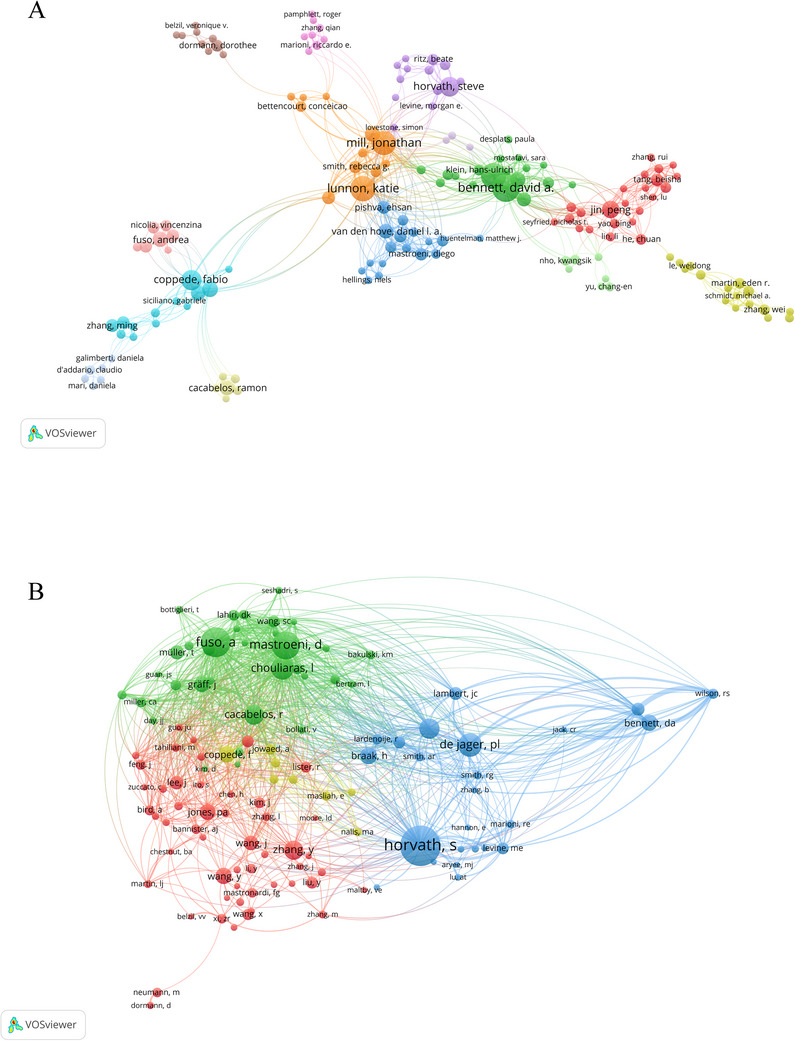
Analysis of authors and co‐cited authors in the field of methylation research related to neurodegenerative diseases. (A) Network visualization map of authors with five or more publications. The size of the nodes represents the number of articles. (B) The network visualization of co‐cited authors with a minimum of 100 citations. Each node represents a co‐cited author, with node size indicating their citations, and the lines between authors representing their collaborations.

The bibliometric analysis revealed that 16,271 authors contributed to publishing in the field of methylation research related to neurodegenerative diseases. As shown in Table 3, Bennett, David A (*n* = 44), ranked first in terms of publication count, followed by De Jager, Philip L. (*n* = 38), Lunnon, Katie (*n* = 38), and Mill, Jonathan (*n* = 35). In addition, Horvath Steve's work stands out with the highest citation count of 3698, indicating not only a significant impact within the field but also a relatively high study quality. In terms of co‐cited authors, a total of 105,047 researchers constitute the co‐cited authors of publications in the field; the top 10 authors have each been cited over two hundred times, with Horvath Steve being the most cited author, leading the list with 635 citations, followed by Fuso A. with 463 citations and Mastroeni D. with 440 citations. Additionally, we used VOSviewer to draw a network map based on the collaboration between authors. A threshold value of 5 was set to construct an author collaboration network, in which Bennett, David A. had the highest TLS (TLS = 159) and had successfully collaborated with 39 authors (Figure 5A). Different clusters are shown in different colors, and authors within the same cluster work more closely together. Furthermore, setting a threshold of 100 for the co‐cited authors' network, Figure 5B displays the outcomes, highlighting 116 authors with more than 100 co‐citations. Each node represents a co‐cited author, with node size indicating their citations, and the lines between authors representing their collaborations.

**TABLE 3:** The top 10 authors and co‐cited authors in the field of methylation research related to neurodegenerative diseases.

Rank	Author	Count	Citation	Total link strength	Co‐cited author	Citation	Total link strength
1	Bennett, David A.	44	2929	159	Horvath, S.	635	6828
2	De Jager, Philip L.	38	1692	141	Fuso, A.	463	6367
3	Lunnon, Katie	38	1830	136	Mastroeni, D.	440	7624
4	Mill, Jonathan	35	1741	138	De Jager, P. L.	374	4923
5	Coppede, Fabio	27	1094	62	Chouliaras, L.	352	6407
6	Horvath, Steve	25	3698	50	Lunnon, K.	316	4392
7	Jin, Peng	21	591	62	Cacabelos, R.	306	4806
8	Jagodic, Maja	20	383	83	Zhang, Y.	304	2915
9	Stoccoro, Andrea	18	580	57	Braak, H.	268	2609
10	Wuellner, Ullrich	18	840	36	Bennett, D. A.	260	4761

### 3.6 Analysis of Co‐Cited References

In this study, we conducted a reference analysis to gain insights into the progression of methylation research related to neurodegenerative diseases. The highly co‐cited literature plays a crucial role as a foundational cornerstone in this investigative domain, offering a wealth of references and delivering profound insights. Among these, the top 10 references in terms of both citations and co‐citations are presented in Tables 4 and 5. These top ten most cited references, which received between 616 and 1519 citations, were published between 2007 and 2019. The article titled “An epigenetic biomarker of aging for lifespan and health span” (Levine et al. 2018) is the most cited, having been co‐cited 1519 times, followed by “Large‐scale meta‐analysis of genome‐wide association data identifies six new risk loci for Parkinson's disease (Nalls et al. 2014)” with 1424 citations and “Identification of novel risk loci, causal insights, and heritable risk for Parkinson's disease: a meta‐analysis of genome‐wide association studies (Nalls et al. 2019)” with 1193 citations. Furthermore, the top three co‐cited references were “Alzheimer's Disease: Early Alterations in Brain DNA Methylation at ANK1, BIN1, RHBDF2, and Other Loci (De Jager et al. 2014)” with 304 citations; “Methylomic Profiling Implicates Cortical Deregulation of ANK1 in Alzheimer's Disease (Lunnon et al. 2014)” with 214 citations; and “DNA Methylation Age of Human Tissues and Cell Types (Horvath 2013)” with 213 citations.

**TABLE 4:** The top 10 most‐cited documents globally.

Rank	Paper	Source	Year	Total Citations	TC per Year	DOI
1	An epigenetic biomarker of aging for lifespan and healthspan	*Aging‐US*	2018	1519	217.00	10.18632/aging.101414
2	Large‐scale meta‐analysis of genome‐wide association data identifies six new risk loci for Parkinson's disease	*Nature Genetics*	2014	1424	129.45	10.1038/ng.3043
3	Identification of novel risk loci, causal insights, and heritable risk for Parkinson's disease: a meta‐analysis of genome‐wide association studies	*Lancet Neurology*	2019	1193	198.83	10.1016/S1474‐4422(19)30320‐5
4	Alzheimer's disease: pathogenesis, diagnostics, and therapeutics	*International Journal of Nanomedicine*	2019	715	119.17	10.2147/IJN.S200490
5	Arginine Methylation: The Coming of Age	*Molecular Cell*	2017	699	87.38	10.1016/j.molcel.2016.11.003
6	Religious Orders Study and Rush Memory and Aging Project	*Journal of Alzheimer's Disease*	2018	674	96.29	10.3233/JAD‐179939
7	Human autoimmune diseases: a comprehensive update	*Journal of Internal Medicine*	2015	672	67.20	10.1111/joim.12395
8	Alzheimer's disease: early alterations in brain DNA methylation at ANK1, BIN1, RHBDF2, and other loci	*Nature Neuroscience*	2014	663	60.27	10.1038/nn.3786
9	Residue‐by‐Residue View of In Vitro FUS Granules that Bind the C‐Terminal Domain of RNA Polymerase II	*Molecular Cell*	2015	619	61.90	10.1016/j.molcel.2015.09.006
10	DNA hypomethylation and human diseases	*Biochimica et Biophysica Acta*	2007	616	34.22	10.1016/j.bbcan.2006.08.007

**TABLE 5:** The top 10 most co‐cited references.

Rank	Cited References	Source	Year	Citations	Total link strength	DOI
1	Alzheimer's disease: early alterations in brain DNA methylation at ANK1, BIN1, RHBDF2, and other loci	*Nature Neuroscience*	2014	304	2986	10.1038/NN.3786
2	Methylomic profiling implicates cortical deregulation of ANK1 in Alzheimer's disease	*Nature Neuroscience*	2014	214	2428	10.1038/NN.3782
3	DNA methylation age of human tissues and cell types	*Genome Biology*	2013	213	1777	10.1186/GB‐2013‐14‐10‐R115
4	Age‐specific epigenetic drift in late‐onset Alzheimer's disease	*PLOS One*	2008	184	2380	10.1371/JOURNAL.PONE.0002698
5	Methylation regulates alpha‐synuclein expression and is decreased in Parkinson's disease patients' brains	*Journal of Neuroscience*	2010	169	1879	10.1523/JNEUROSCI.6119‐09.2010
6	Consistent decrease in global DNA methylation and hydroxymethylation in the hippocampus of Alzheimer's disease patients	*Neurobiology of Aging*	2013	166	2421	10.1016/J.NEUROBIOLAGING.2013.02.021
7	Epigenetic changes in Alzheimer's disease: decrements in DNA methylation	*Neurobiology of Aging*	2010	155	2316	10.1016/J.NEUROBIOLAGING.2008.12.005
8	S‐adenosylmethionine/homocysteine cycle alterations modify DNA methylation status with consequent deregulation of PS1 and BACE and beta‐amyloid production	*Molecular and Cellular Neuroscience*	2005	141	1621	10.1016/J.MCN.2004.09.007
9	CpG demethylation enhances alpha‐synuclein expression and affects the pathogenesis of Parkinson's disease	*PLOS One*	2010	141	1552	10.1371/JOURNAL.PONE.0015522
10	Global changes in DNA methylation and hydroxymethylation in the Alzheimer's disease human brain	*Neurobiology of Aging*	2014	134	1947	10.1016/J.NEUROBIOLAGING.2013.11.031

**FIGURE 6:**
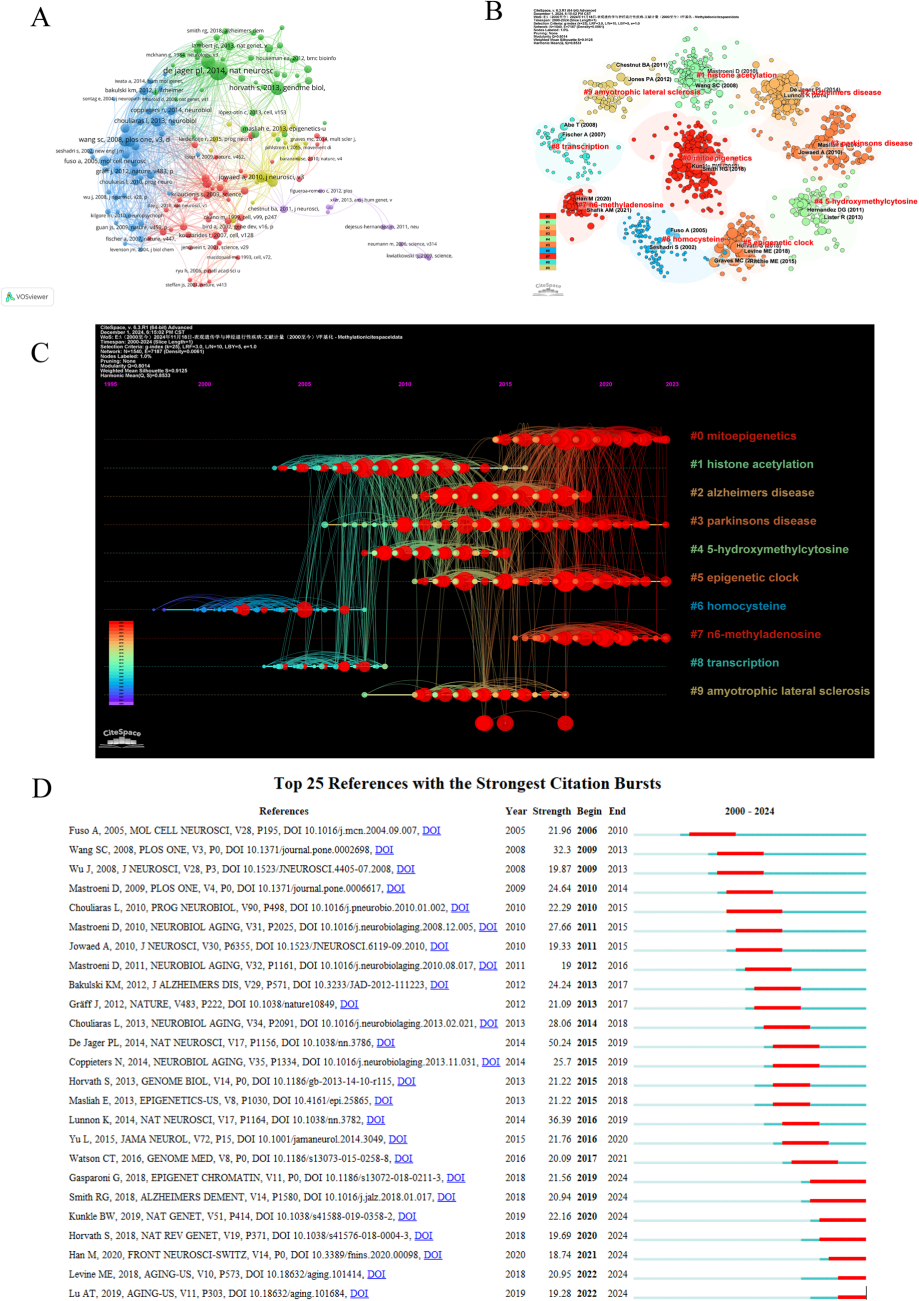
An analysis of co‐cited references in the field of methylation research related to neurodegenerative diseases. (A) The network map of co‐cited references shows that of the 170,172 references, 158 were cited at least 50 times. The size of the nodes represents the co‐citation frequency of the references. (B) The top 10 clusters from the co‐cited references analysis are presented. Different colors represent different clusters. (C) Timeline distribution of the top 10 clusters. The larger nodes denote a higher reference count. Nodes on the left represent earlier references, while those on the right represent more recent ones. (D) Top 25 references with the strongest citation bursts.

Figure 6A presents the network diagram of co‐citation references. Furthermore, a co‐citation analysis of 170,172 references was conducted, with 158 references co‐cited over 50 times. The visualization is divided into five distinct clusters, each represented by a unique color that corresponds to different sets of references. The red cluster encompasses 45 references, the green cluster contains 41, the blue cluster comprises 40, the yellow cluster consists of 18, and the purple cluster includes 14 references. According to TLS, the top three co‐citation references were as follows: “Alzheimer's disease: Early alterations in brain DNA methylation at ANK1, BIN1, RHBDF2, and other loci (De Jager et al. 2014)” (TLS = 2986), “Methylomic profiling implicates cortical deregulation of ANK1 in Alzheimer's disease (Lunnon et al. 2014)” (TLS = 2428), and “Consistent decrease in global DNA methylation and hydroxymethylation in the hippocampus of Alzheimer's disease patients (Chouliaras et al. 2013)” (TLS = 2421).

Reference clustering analysis, a statistical method that groups similar literature based on specific characteristics, can reveal the structure, trends, and evolution of knowledge in a research field when integrated with the timeline view of co‐citation data. This approach also tracks the historical development and trends of research topics, identifies when certain works or authors become influential, and illustrates how the focus of research shifts over time within a particular domain. Figure 6B,C present the co‐citation network clustering and its timeline view, highlighting 10 primary clusters: Cluster #0 for mitoepigenetics, Cluster #1 for histone acetylation, Cluster #2 for Alzheimer's disease, Cluster #3 for Parkinson's disease, Cluster #4 for 5‐hydroxymethylcytosine, cluster #5 for the epigenetic clock, cluster #6 for homocysteine, cluster #7 for N6‐methyladenosine, cluster #8 for transcription, and cluster #9 for amyotrophic lateral sclerosis.

In addition, we identified the top 25 references with the strongest citation bursts, reflecting the frequency with which particular studies are cited. These references represent literature that has been closely followed by scholars in the field of methylation research related to neurodegenerative diseases over a certain period, indicating that they have been widely cited by other studies and have received particular attention. As shown in Figure 6D, the article entitled “Alzheimer's disease: early alterations in brain DNA methylation at ANK1, BIN1, RHBDF2 and other loci (De Jager et al. 2014)” written by Philip L. De Jage has the highest burst strength (50.24), followed by “Methylomic profiling implicates cortical deregulation of ANK1 in Alzheimer's disease (Lunnon et al. 2014)” (36.39) and “Age‐specific epigenetic drift in late‐onset Alzheimer's disease (S. C. Wang et al. 2008)” (32.30). These papers emphasize the important role of DNA methylation in the early development of Alzheimer's disease and identify methylation changes at specific genes and loci that may be related to the progression and development of neurodegenerative diseases.

### 3.7 Analysis of Keywords

**FIGURE 7:**
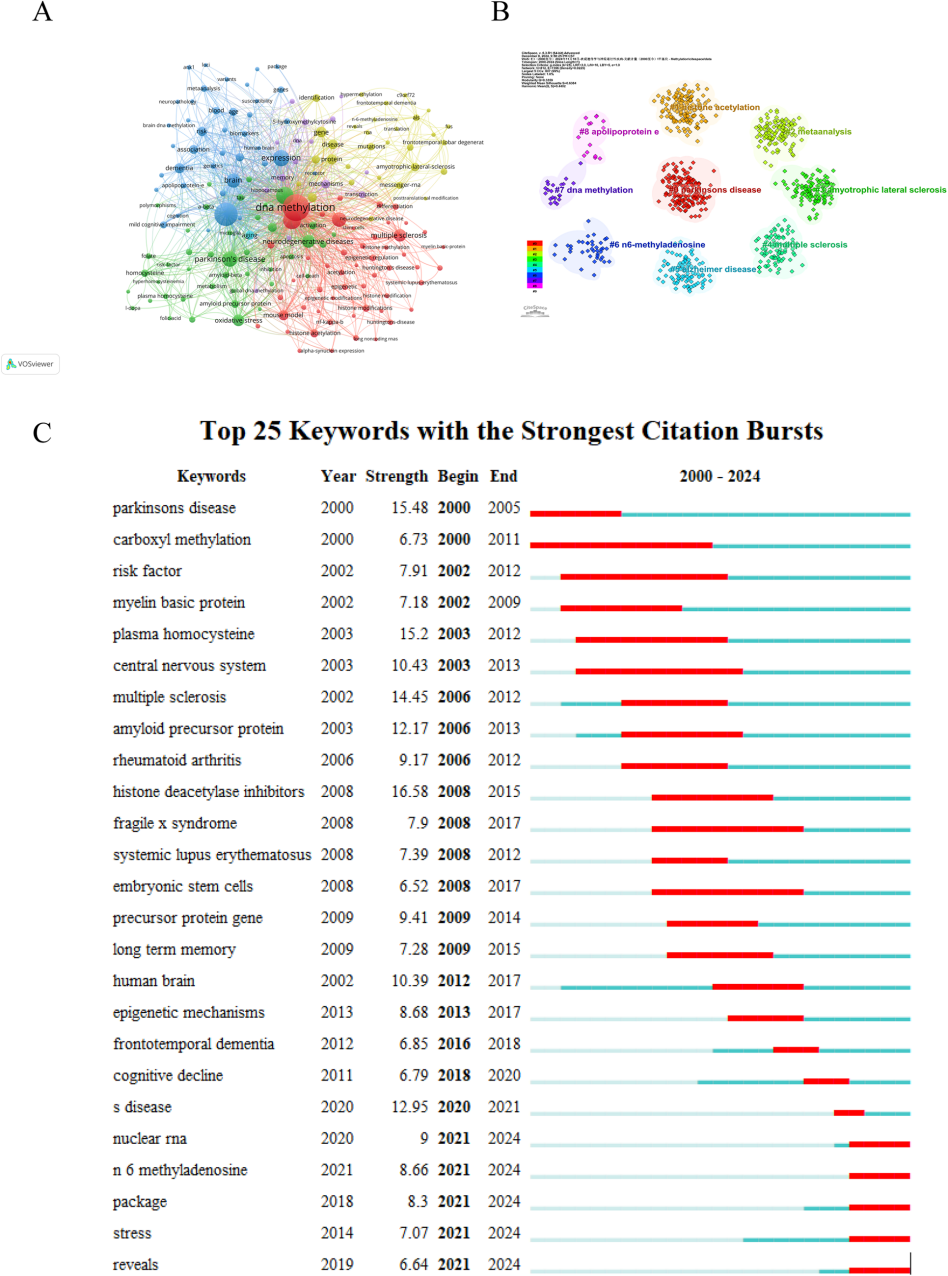
Cluster analysis of keywords from 2000 to 2024. (A) The network visualization map presents a co‐occurrence and clustering analysis of frequent keywords, highlighting those that appeared more than 50 times. (B) Analysis of keyword cluster. Different colors represent different clusters. (C) Top 25 keywords with the strongest citation bursts.

Keywords encapsulate the core concepts of a study, and analyzing their co‐occurrence provides insights into the relationships among key terms within the field of methylation research related to neurodegenerative diseases. Utilizing VOSviewer, we visualized an extensive network of keywords that co‐occurred frequently. Specifically, 181 keywords were selected for the network from a total of 12,051, based on the criterion of appearing more than 50 times in the dataset, as illustrated in Figure 7A. The top 10 keywords were “DNA methylation,” “Alzheimer's disease,” “epigenetics,” “methylation,” “expression,” “Parkinson's disease,” “gene‐expression,” “brain,” “neurodegenerative diseases,” and “multiple sclerosis.”

A cluster analysis of keywords was performed to explore the central knowledge framework in the field of study, resulting in the division of the study into 9 clusters, as illustrated in Figure 7B: Cluster #0 focuses on Parkinson's disease, Cluster #1 on histone acetylation, Cluster #2 on meta‐analysis, Cluster #3 on amyotrophic lateral sclerosis, Cluster #4 on multiple sclerosis, Cluster #5 on Alzheimer's disease, Cluster #6 on N6‐methyladenosine, Cluster #7 on DNA methylation, and Cluster #8 on apolipoprotein E. Each cluster's size indicates the frequency with which the keywords appear in the literature, while the density of connecting links between keywords underscores the intricate and complex relationships within the research domain. This cluster analysis not only reveals the breadth of research topics but also the depth of investigation in each area, offering a snapshot of the current state of research and potential avenues for future exploration.

Keyword bursts, which represent a significant and abrupt increase in the frequency of specific terms within a research field over a defined timeframe, can be indicative of emerging trends or new directions in academic inquiry. Keyword bursts, as depicted in Figure 7C, highlight significant trends in methylation research related to neurodegenerative diseases from 2000 to 2024. The most frequently mentioned keywords include histone deacetylase inhibitors, Parkinson's disease, plasma homocysteine, multiple sclerosis, Alzheimer's disease, amyloid precursor protein, central nervous system, human brain, and so on. These keywords provide a snapshot of the dynamic research landscape in neurodegenerative diseases, particularly emphasizing the role of methylation and epigenetic modifications in disease pathogenesis and potential therapeutic development.

## Discussion

The bibliometric analysis indicated a notable increase in research publications concerning methylation and neurodegenerative diseases, with a total of 3284 articles identified over the last two decades. This trend suggests a growing interest and investment in exploring the epigenetic mechanisms that contribute to these conditions. However, it is important to note that while bibliometric trends can reflect research activity, they do not directly establish causation or clinical impact. This burgeoning field not only appears to be a promising area for funding and resource allocation but may also be linked to improvements in clinical outcomes for neurodegenerative diseases. As the volume of research outputs rises, it becomes crucial to investigate how this trend might shape future funding initiatives and collaborative efforts, as well as its connection to advancements in treatment protocols. Additionally, the increasing number of publications may signal technological progress in research methodologies, which could encourage interdisciplinary collaborations that enhance the quality and significance of findings in this vital area of study.

The geographical distribution of research contributions shows a significant concentration of efforts in the USA, China, and the United Kingdom, which together account for over 66% of total publications. The USA stands out not only for its high volume of publications but also for its strong citation impact, highlighting its leading role in advancing methylation research. Interestingly, Canada exhibits a higher average citation rate despite producing fewer publications than China, indicating possible differences in research quality, levels of international collaboration, and a focus on high‐impact studies. These trends underscore the need for enhanced global knowledge exchange and resource sharing to improve the collective response to neurodegenerative diseases. As emerging nations increasingly participate in this field, their contributions may introduce fresh perspectives and innovative therapeutic approaches, further underscoring the importance of methylation studies in tackling neurodegenerative diseases. To address existing disparities, future funding initiatives should focus on encouraging international partnerships, especially with emerging research hubs, to foster equitable collaboration and expedite breakthroughs in this vital area.

Analysis of institutional contributions reveals that leading institutions such as the University of California System and Harvard University significantly advance research on methylation and its links to neurodegenerative diseases. Their impressive publication and citation rates reflect a robust research environment that fosters innovation and collaboration. A collaborative network map further demonstrates the interconnections among these institutions, underscoring the importance of partnerships in enhancing research productivity and encouraging interdisciplinary approaches. The relationship between an institution's reputation and its funding opportunities is crucial for attracting top talent and resources. By identifying key players within this collaborative framework, we can better direct future research efforts, which may lead to important breakthroughs in understanding the molecular mechanisms underlying neurodegenerative diseases.

The analysis of journals in this field indicates that high‐impact platforms such as the “*International Journal of Molecular Sciences*” and the “*Journal of Alzheimer's Disease*” play a crucial role in disseminating research findings. The prevalence of publications in Q1 and Q2 journals underscores the high quality of research being conducted and reflects the evolving landscape of academic publishing in this domain. Moreover, co‐citation analysis reveals the interconnectedness of research topics, suggesting that journals frequently cited together often share thematic relevance. By grasping these publication trends, researchers can pinpoint appropriate platforms for sharing their findings, ensuring that significant insights reach the intended audiences. Additionally, as the field advances, the growing significance of open access and collaborative publishing may enhance journal visibility and citation rates, potentially influencing future research directions in the areas of methylation and neurodegenerative diseases.

The keyword analysis has shed light on research topics related to methylation and neurodegenerative diseases, highlighting key areas of interest such as “DNA methylation,” “Alzheimer's disease,” and “epigenetics.” This analysis underscores the connections among these themes and suggests opportunities for interdisciplinary collaboration. By examining these trends, researchers can better establish future research priorities, as it provides a strategic framework to navigate the complexities of studying neurodegenerative diseases. Additionally, the analysis points out potential pathways for innovative methodologies that combine various scientific disciplines. As new keywords emerge, they may indicate shifts in research focus, which can help develop novel therapeutic strategies and improve our understanding of the pathophysiological mechanisms that underlie neurodegenerative diseases.

The study offers valuable insights into the role of methylation in neurodegenerative diseases; however, it does have certain limitations. Primarily, it is based on bibliometric analysis, which may overlook unpublished or ongoing research, thereby limiting the generalizability of its findings. Furthermore, by concentrating solely on publication metrics, the study fails to address the clinical implications of these research trends, resulting in a gap in understanding how these findings may influence therapeutic or diagnostic advancements in the field.

This bibliometric analysis highlights the growing emphasis on DNA methylation research related to neurodegenerative diseases, revealing key trends, major contributors, and collaborative networks in this area. The results underscore the need for ongoing investigation into the molecular mechanisms that drive these diseases and point to the potential benefits of collaborative efforts in creating new therapeutic approaches. Bibliometric analysis serves not only to pinpoint research trends and hotspots but also to provide practical insights for laboratory work. By integrating bibliometric findings with experimental research, scientists can design more focused experiments, refine research methods, and bridge the divide between fundamental discoveries and clinical applications. This study not only outlines the evolving field of methylation research but also presents practical strategies to align computational advancements with laboratory and clinical needs. By focusing on impactful subfields and fostering translational collaborations, the research community can accelerate the development of precision therapies for neurodegenerative diseases.

## Author Contributions

**Jianwei Xu**: project administration, conceptualization, writing–original draft, writing–review and editing. **Junhou Lu**: software, writing–original draft, writing–review and editing. **Tao Kuang**: validation, formal analysis, writing–review and editing. **Dongyan Wang**: resources, visualization, writing–review and editing. **Zhihui Dong**: writing–review and editing, resources, formal analysis. **Maoqiong Chen**: formal analysis, writing–review and editing, methodology. **Can Liu**: data curation, methodology, writing–review and editing. **Guo Ge**: investigation, writing–review and editing, writing–original draft, resources. **Tianhong Yuan**: validation, writing–review and editing, methodology, supervision. **Zhen Qin**: data curation, funding acquisition, methodology, supervision, writing—original draft, writing–review and editing.

## Conflicts of Interest

The authors declare no conflicts of interest.

## Peer Review

The peer review history for this article is available at https://publons.com/publon/10.1002/brb3.70732.

## Data Availability

The data that support the findings of this study are available from the corresponding author upon reasonable request.
